# Higher healing rate after meniscal repair with concomitant ACL reconstruction for tears located in vascular zone 1 compared to zone 2: a systematic review and meta-analysis

**DOI:** 10.1007/s00167-022-06862-2

**Published:** 2022-01-24

**Authors:** L. M. Gerritsen, T. J. N. van der Lelij, P. van Schie, M. Fiocco, E. R. A. van Arkel, R. G. Zuurmond, S. Keereweer, P. B. A. A. van Driel

**Affiliations:** 1 Department of Orthopedic Surgery, Leiden University Medical Center, Post zone J10-R83, P.O. Box 9600, 2300 RC Leiden, The Netherlands; 2 Mathematical Institute Leiden University, Leiden, The Netherlands; 3 Department of Biomedical Data Science, Medical Statistics Section, Leiden University Medical Center, Leiden, The Netherlands; 4 Department of Orthopedic Surgery, Haaglanden Medical Center, The Hague, The Netherlands; 5 Department of Orthopedic Surgery, Isala, Zwolle, The Netherlands; 6 Department of Otorhinolaryngology and Head and Neck Surgery, Erasmus MC Cancer Institute, University Medical Center Rotterdam, Rotterdam, The Netherlands

**Keywords:** Meniscal repair, Meniscus, Vascularization, Arthroscopy, Knee

## Abstract

**Purpose:**

The purpose of this study was to determine and compare the percentage of completely healed meniscal tears after arthroscopic repair combined with anterior cruciate ligament reconstruction (ACLR) for the different vascular zones of the meniscus.

**Methods:**

PubMed, Embase, Web of Science, Cochrane library and Emcare were searched on 19 May 2020 for articles reporting healing rates after arthroscopic meniscal repair with concomitant ACLR for the different meniscal vascular zones as assessed by second-look arthroscopy. Data on meniscal tears were extracted as located in zones 1, 2 or 3, according to the Cooper classification. Studies were graded in quality using a modified Newcastle–Ottawa Scale. Pooled analyses were performed utilizing a random-effects model. Meta-analyses were performed using R version 3.6.2 and SPSS statistical software version 25.0. The study was registered with PROSPERO (ID:CRD42020176175).

**Results:**

Ten observational cohort studies met the inclusion criteria, accounting for 758 meniscal tear repairs in total. The pooled overall proportion of healing was 78% (95% CI 72–84%). The mean weighted proportion of healing was 83% (95% CI 76–90%) for studies (*n* = 10) reporting zone 1 tears and 69% (95% CI 59–79%) for studies (*n* = 9) reporting zone 2 tears. No study reported healing rates for zone 3 tears. The pooled overall odds ratio was 2.5 (95% CI 1.00−6.02), indicating zone 1 tears as 2.5 times more likely to heal than zone 2 tears.

**Conclusion:**

This study demonstrates that meniscal tears localized in vascular zone 1 were more likely to heal than those in zone 2.

**Level of evidence:**

IV.

**Supplementary Information:**

The online version contains supplementary material available at 10.1007/s00167-022-06862-2.

## Introduction

Meniscal lesions are one of the most common injuries in orthopaedic surgery and can be surgically treated by repair or by either partial, subtotal or total meniscectomy [17]. When possible, a repair is preferred because (partial) meniscectomy is associated with osteoarthritis in the long term [25]. A wide range of repair techniques, meniscal devices and biological augmentation are described in current literature [35]. Despite careful patient selection and improved surgical techniques, meniscal repairs have up to 30% clinical failure rates [12, 14, 21]. Several factors are reported to affect the clinical healing rate of meniscal tears after repair, such as type of meniscal tear, time of repair after injury, and concomitant ACL reconstruction (ACLR) [17, 21, 31, 35, 36]. The meniscal healing process is based on two fundamental principles: a solid primary fixation and a well-functioning biological process of cicatrization, where the presence of vascularization is thought to play a major role [2, 4, 35].

In 1982, Arnoczky et al. reported that the extent of vascular penetration in adults ranged from 10 to 30% for the medial meniscus and 10–25% for the lateral meniscus [2]. A tear can occur in a vascularized part as well as in an avascular area, and the Cooper classification is commonly used to describe the specific tear location. This classification includes an arbitrary division of the meniscus into thirds, both longitudinally and radially [7]. Longitudinal zones are divided based on vascularization and are often referred to as ‘‘red-red” zone (zone 1), ‘‘red-white” zone (zone 2) and ‘‘white-white” zone (zone 3). The ‘‘red-red” zone is the most vascularized part of the meniscus and the ‘‘white-white” zone the avascular part. The ‘‘red-white” zone separates these two and is considered partially vascularized [2, 5, 18]. For an accurate evaluation of the influence of vascularization on healing and comparison of outcomes after meniscal repair, it is important to classify tears according to their vascular zones [32].

Pre-clinical studies have shown that healing of meniscal tears is inherently bound to vascularity of the surrounding tissue [2, 8, 34]. In two comprehensive reviews about meniscal repairs, Woodmass et al. and Vaquero-Picado et al. emphasized the importance of the meniscal blood supply on clinical healing rate after meniscal repair [31, 35]. Nevertheless, the literature is ambiguous, as Yeo et al. could not support increased tear vascularity or a smaller rim width as a predictive factor for tear healing [36]. Furthermore, although meniscal tears in the avascular zone theoretically have less healing capacity, some studies report successful repair of meniscal tears in the avascular zone, as defined by reoperation rate [15].

Completely healed meniscal tears after repair might result in functionally superior menisci and consequently better long-term outcomes than incomplete or non-healed tears. To assess healing, most studies look at clinical outcomes after meniscal repair, such as Patient Reported Outcome Measures (PROMs) or clinical tests, or imaging modalities such as MR imaging. The sensitivity, specificity and accuracy of clinical outcomes and different imaging modalities to determine biologically complete healing remains limited [11, 16, 17, 19, 27]. The most reliable technique to evaluate complete meniscal healing is second-look arthroscopy [17]. The aim of this study is to systematically review the literature on healing rates after meniscal repair for each vascular zone, as assessed by second-look arthroscopy, and perform a meta-analysis. By assessing the effect of specific vascular zones on healing rates, patient-specific chances for successful meniscal repair could be determined more precisely. In order to increase comparability across studies, this review only includes studies with concomitant ACLR using second-look arthroscopy to assess outcome. It was hypothesized that repaired meniscal tears in zone 1 are more likely to heal than zone 2 and zone 3 tears.

## Materials and methods

This systematic review and meta-analysis was conducted following the Preferred Reporting Items for Systematic Reviews and Meta-Analyses (PRISMA) statement and was registered with PROSPERO prior to the screening of studies [24].

### Systematic review

A search strategy was constructed by an experienced librarian (JS). PubMed, Embase, Web of Science, Cochrane library and Emcare were searched for publications up to 19 May 2020, without restriction of publication date. The search included two components: ‘‘Meniscal tear’’ OR ‘’Meniscal repair’’ and ‘‘Healing’’ OR ‘‘Success’’ OR ‘‘Failure’’ (see Appendix I for the complete search strategy). A search term for ‘‘vascularization’’ was not combined in the search to avoid missing relevant studies, as a wide variety of words is used in literature to described the vascular zones of the meniscus (e.g. rim width, red–red/red–white/white–white zone, peripheral/middle/central third, avascular/vascular zone, Cooper zone 1/2/3). Articles in English and Dutch were included to ensure consistent high quality of data extraction and quality assessment. Only peer-reviewed studies were considered eligible for inclusion.

### Inclusion and exclusion criteria

Studies describing healing rate after meniscal repair with ACLR for specific vascular zones were included. Only studies that confirmed a healed meniscus by second-look arthroscopy were included. The excluded studies were the ones that: did not describe healing or success rates for vascular zones after meniscal repair; did not perform concomitant ACLR; did not assess healing status by second-look arthroscopy; did not include human patients; included < 10 patients; or were not written in English or Dutch.

### Screening

**Fig. 1:**
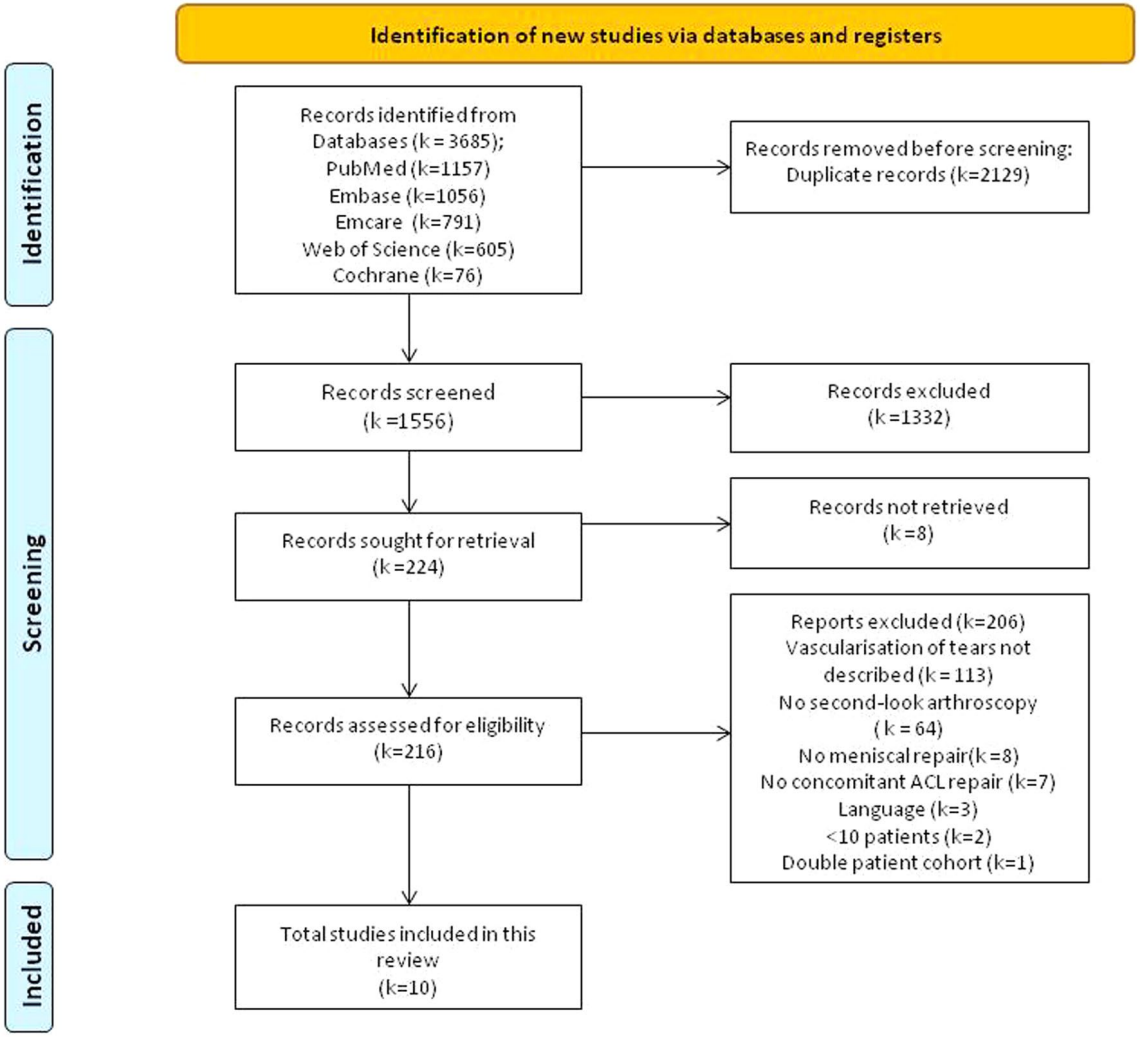
Flowchart of literature selection

All titles and abstracts were screened independently by two reviewers (MG&TvL). Subsequently, full-text articles were reviewed by the same reviewers (MG&TvL). Discrepancies were resolved through discussion. Two additional reviewers (PvS&PvD) were available when consensus could not be reached. A PRISMA (statement 2020) flowchart is provided in Fig. 1 [24].

#### Data extraction

Data were extracted by both reviewers (MG&TvL) independently using a prespecified data extraction sheet in Microsoft Excel 2016 (Microsoft, Redmond, WA, USA). Data on healing rates after meniscal repair for different vascular zones were extracted. Meniscal healing assessed with second-look arthroscopy is generally classified as ‘‘complete’’, ‘‘incomplete/partial’’ or ‘‘no healing’’, according to the criteria of Morgan et al. [20]. Since we aimed to review complete healing after meniscal repair, we classified data on “incomplete” and “partial” healing as failure. For multiple publications involving the same cohort, data were extracted from the study with the most comprehensive description of the cohort. Furthermore, sample size, inclusion period, surgical technique, inclusion criteria, indication for second-look, mean age of cohort and gender were extracted. Data on vascular region were extracted following the classification guidelines of the International Society of Arthroscopy Knee Surgery and Orthopaedics Sports Medicine [1]. Following this classification, zone 1 tears have a rim width < 3 mm measured from the meniscocapsular junction and zone 2 a rim width of 3 to < 5 mm. However, studies not specifying rim width, but classifying tears as red–red and red–white, were extracted as zone 1 and zone 2, respectively.

### Assessment of risk of bias

Risk of bias was assessed independently by both reviewers (MG&TvL) using a modified version of the Newcastle–Ottawa scale (NOS) (see Appendix II). The NOS is a tool for the quality assessment of non-randomized studies [33]. Studies with a NOS score of 0–3 points were considered to be of *low* quality, 4–6 points of moderate quality and > 7 points of high quality.

### Data and statistical analysis

**Fig. 2:**
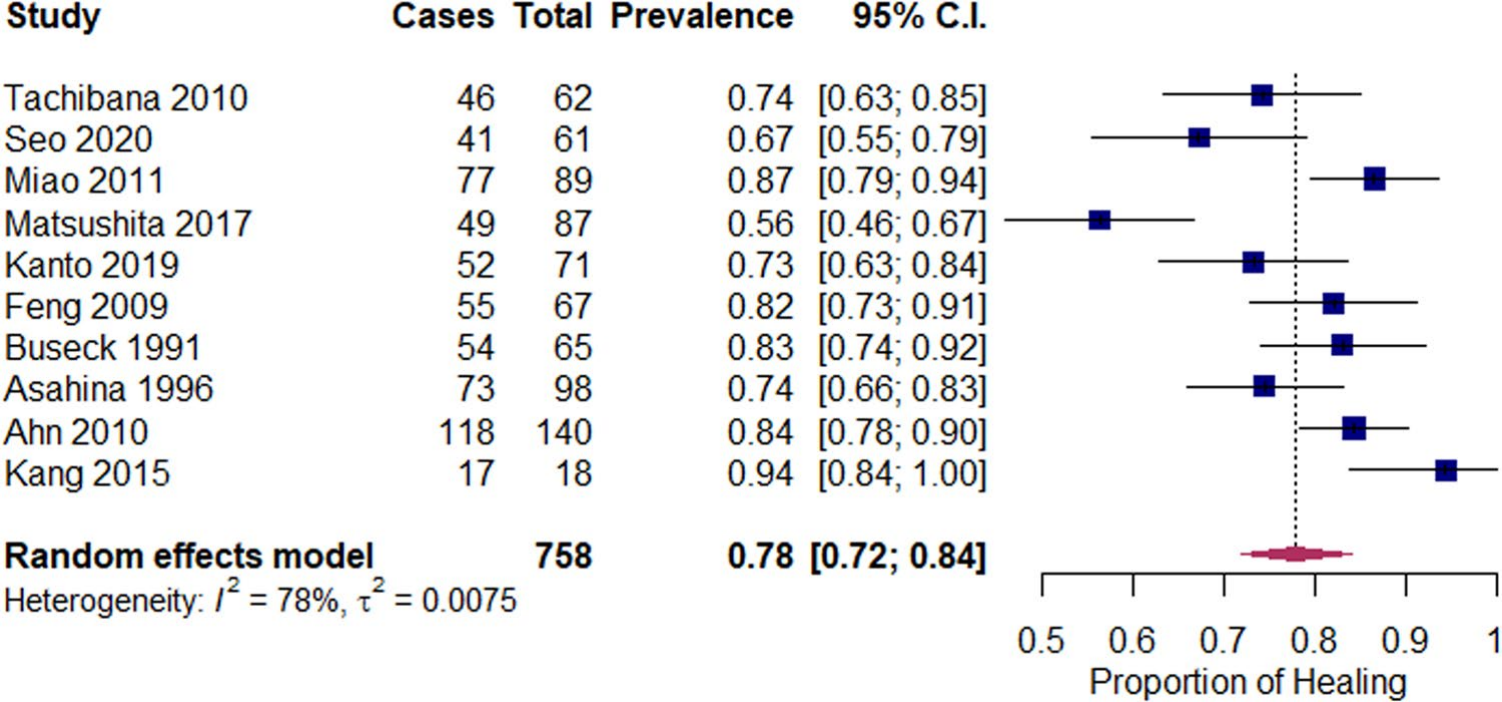
Forest plot for proportion of completely healed tears after meniscal repair in all vascular zones

**Fig. 3:**
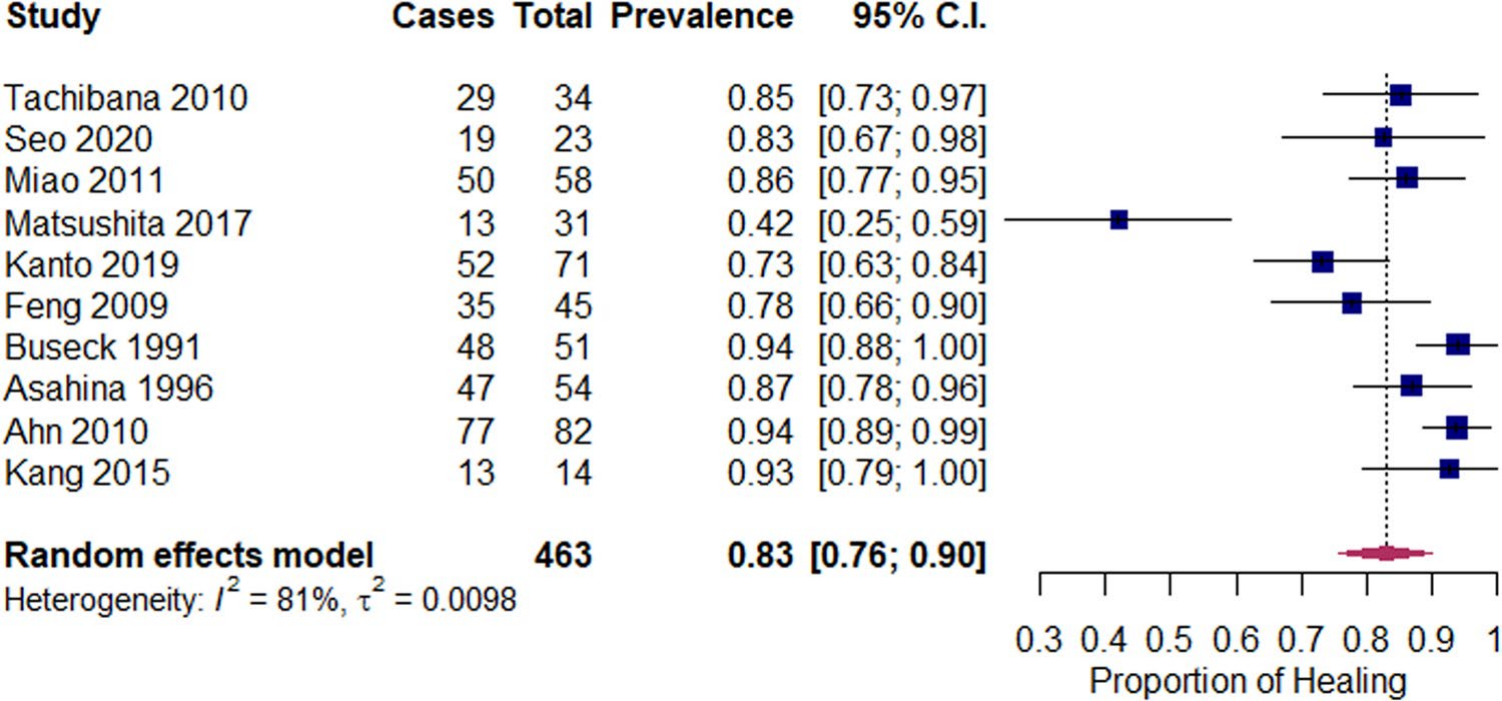
Forest plot for proportion of completely healed zone 1 tears after meniscal repair

**Fig. 4:**
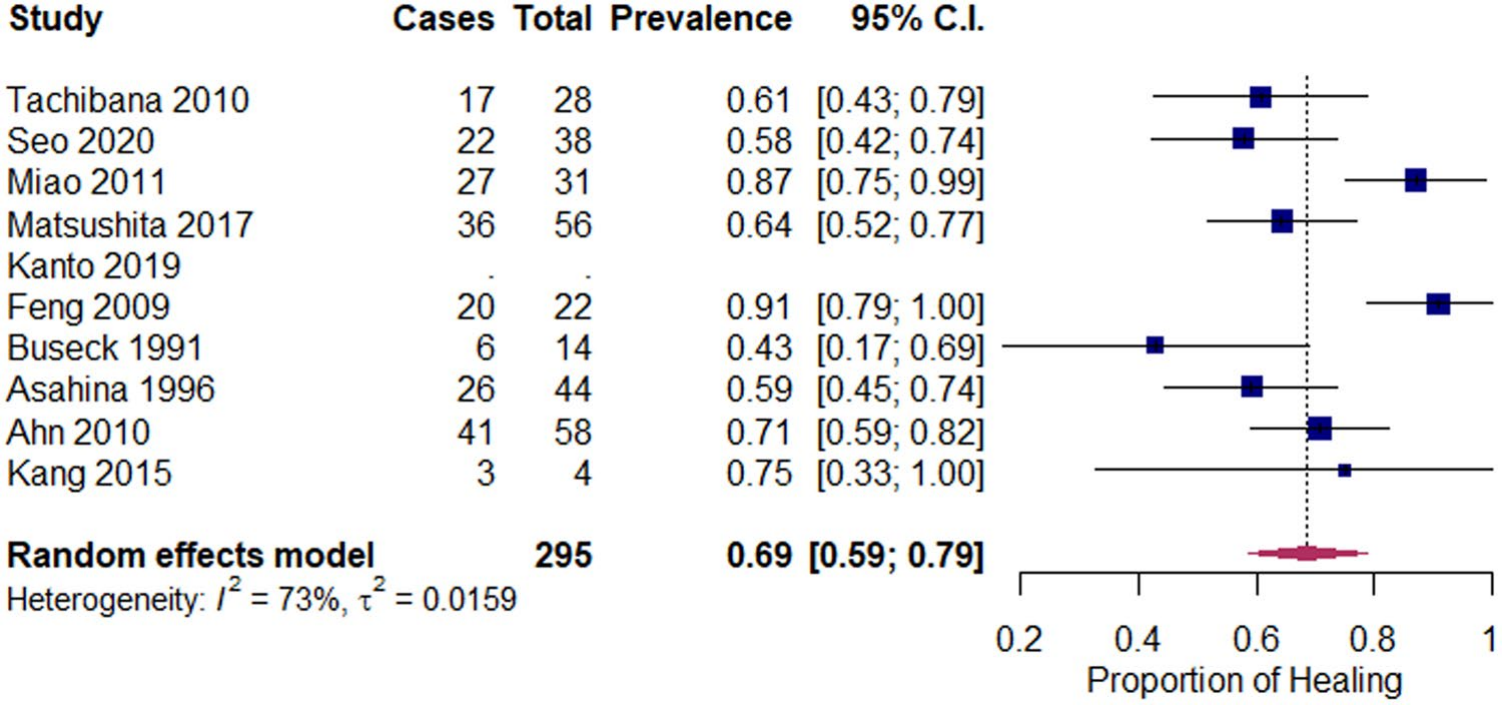
Forest plot for proportion of completely healed zone 2 tears after meniscal repair

The inclusion criteria in this review were very strict, to gain the most comparable groups of patients at baseline and further allowing data from all studies—regardless of study design (retrospective or prospective)—to be pooled. Random effect models were employed to pool two study-specific measures, proportion and odds ratio to estimate overall effect and its associated confidence intervals (CIs). Inverse variance method, which gives more weight to larger studies, was used to pool outcomes for different studies. Overall effects estimated with a random effects model are reported together in the same forest plots along with their CIs. The sizes of the square boxes on the forest plot are proportional to the total number of patients in the selected trials. An overall test on heterogeneity between studies was performed (value *I*-squared in Figs. 2, 3 and 4). The *I*-squared statistic describes the proportion of variation across studies due to heterogeneity [13]. To estimate the between-study variance as “tau” in the forest plots, DerSimonian-Laird’s method was employed [9]. Treatment success, measured as proportion of healed cases in total cases with accompanying 95% CIs, were used as summary outcome measure for each included study. Two different meta-analyses were performed by considering subgroups based on vascular region of the tear: patients with a meniscal tear in zone 1 and those with a meniscal tear in zone 2. To assess frequencies and normal deviation in study characteristics, the Shapiro–Wilk test was employed. Data were analyzed using package Metafor in R version 3.6.2 (The R Foundation for Statistical Computing Platform) and SPSS statistical software version 25.0 (SPSS Inc., Chicago).

## Results

### Study selection

The search yielded 3685 records, and 1556 remained after removing duplicates. A total of ten articles were considered eligible for inclusion (Appendix IV). Specific details on the study selection process and reasons of exclusion can be found in the PRIMSA flowchart (Fig. 1).

### Quality of studies (risk of bias)

Eight studies were identified as reporting high and two as moderate quality of evidence according to the Newcastle–Ottawa scale (NOS) (Table 1). Further details on the allocated risk of bias score are provided in Appendix III.

**Table 1:** Baseline characteristics of included cohorts (*n* = 10)

Author	Country	Inclusion period	Mean Age (years)	Sample Size	Inclusion [exclusion] criteria	2nd-look indications	Surgical technique	Mean follow-up (months)	Quality of evidence
Ahn et al. 2010	South Korea	1997–2007	30	140	Patients undergoing medial meniscus posterior horn repair and ACLR, presenting with a tear displaceable with a probe, longitudinal tear > 1 cm, tear within 5 mm of meniscocapsular junction or double-longitudinal tears [Exclusion criteria: Isolated meniscal repair and combined patellar tendon–bone (BPTB) graft or double-loop hamstring tendon (LDHT) graft.]	Removal of post-tie washer/screw hardware removed from proximal tibia	All-inside and inside-out	38	High
Seo et al. 2020	South Korea	2012–2015	30	61	Patients with medial or lateral posterior horn tear in red-red or red-white zones and concurrent ACLR [Exclusion criteria: Multiple-ligament injury, meniscectomy, revision ACLR, failed ACLR; no 2nd-look arthroscopy.]	Removal of hardware	All-inside	16	High
Asahina et al. 1996	Japan	1986–1994	23	98	Patients undergoing meniscal repair for unstable full-thickness, vertical, longitudinal tear > 15 mm, with ACLR	Removal of staples	Inside-out	16	High
Buseck et al. 1991	USA	1983–1988	22	65	Patients with meniscal tear undergoing meniscal repair with concurrent with ACLR	Removal of painful tibial hardware or meniscal symptoms	Inside-out	12	High
Feng et al. 2008	China	2002–2006	25	67	Patients with bucket handle tears in red–red and red–white zones without additional complex tears and tissue degeneration and with or without concomitant ACL injuries [Exclusion criteria: Multiple-ligament injuries other than ACL and medial collateral.]	Removal of ACL graft metal interference screws, symptomatic patients or second-stage ACLR	Inside-out and all inside	26	Moderate
Kanto et al. 2019	Japan	2009–2015	24	71	Patients undergoing meniscal repair with concomitant primary ACLR [Exclusion criteria: Concomitant osseous or ligamental surgeries or follow-up < 1 year or lack of 2nd-look arthroscopy results.]	Removal of tibial screw or suspicion of intra-articular lesions such as meniscal and cyclops lesions	All-inside and inside out	15	Moderate
Matsushita et al. 2017	Japan	2004–2012	25	87	Patients with longitudinal tear in red–red zone or red–white zone, or bucket-handle tear with ACLR	Removal of post-screw or because of clinical symptoms	All-inside and inside-out	16	High
Miao et al. 2011	China	2005–2007	25	89	Patients aged < 50 years, with vertical tears < 10 mm in length located in the red zone or red–white zone, concomitant ACLR and no additional injury on the operated knee joint	Removal of staples used to fix the reconstructed ACL	All-inside	25	High
Tachibana et al. 2010	Japan	2005–2008	27	62	Patients with longitudinal or double longitudinal tears in the red–red and red–white zones or unstable torn menisci undergoing meniscal repair concurrent with ACLR	Staged hardware removal after ACLR	All-inside	14	High
Kang et al. 2015	Korea	2010–2012	35	18	Patients with meniscal tears in the red–red and red–white zones undergoing meniscal repair with concomitant ACLR [Exclusion criteria: Isolated meniscal repair or combined knee injuries other than ACL.]	Not specified	All-inside	17	High

### Study characteristics

Ten observational cohort studies were included, three prospective and seven retrospective. Randomized studies were not available for inclusion. No serious adverse events related to meniscal repair were reported in the included studies. Further details on baseline characteristics of the included studies are provided in Table 1.

The ten included studies accounted for 758 arthroscopic meniscal repairs, with a mean sample size of 81 (standard deviation [SD] 35; range 18–140) per cohort. Mean age of participants was 27 years (SD 4; range 23–35 years) and 37% (SD 23%; range 10–67%) were female. Duration of follow-up was provided for all studies and median follow-up time was 20 months (SD 8; range 14–38).

### Proportion of healing after meniscal repair

The pooled proportion of healing was 78% (95% CI 72–84%), with high heterogeneity between studies (*I*^2^ = 78%) (Fig. 2). The healing rates for all studies ranged from 56 to 94%.

#### Zone 1 tears

Ten studies described the outcomes of zone 1 tears. Pooled analysis of 463 zone 1 tears revealed a pooled proportion of healing of 83% (95% CI 76–90%), with reported healing rates per study ranging from 42 to 94% (Fig. 3). The zone 1 subgroup showed high heterogeneity between studies (*I*^2^ = 81%).

#### Zone 2 tears

The pooled data for zone 2 tears included 295 menisci from 9 studies. The pooled proportion of healing was 69% (95% CI 59–79%), with individual studies ranging from 43 to 91% (Fig. 4). Heterogeneity for the proportion of healing for zone 2 tears was 73%.

#### Zone 3 tears

No studies were included describing outcome for specified zone 3 tears after meniscal repair with concomitant ACLR assessed with second-look arthroscopy.

### Effect of vascularization on healing after meniscal repair

**Fig. 5:**
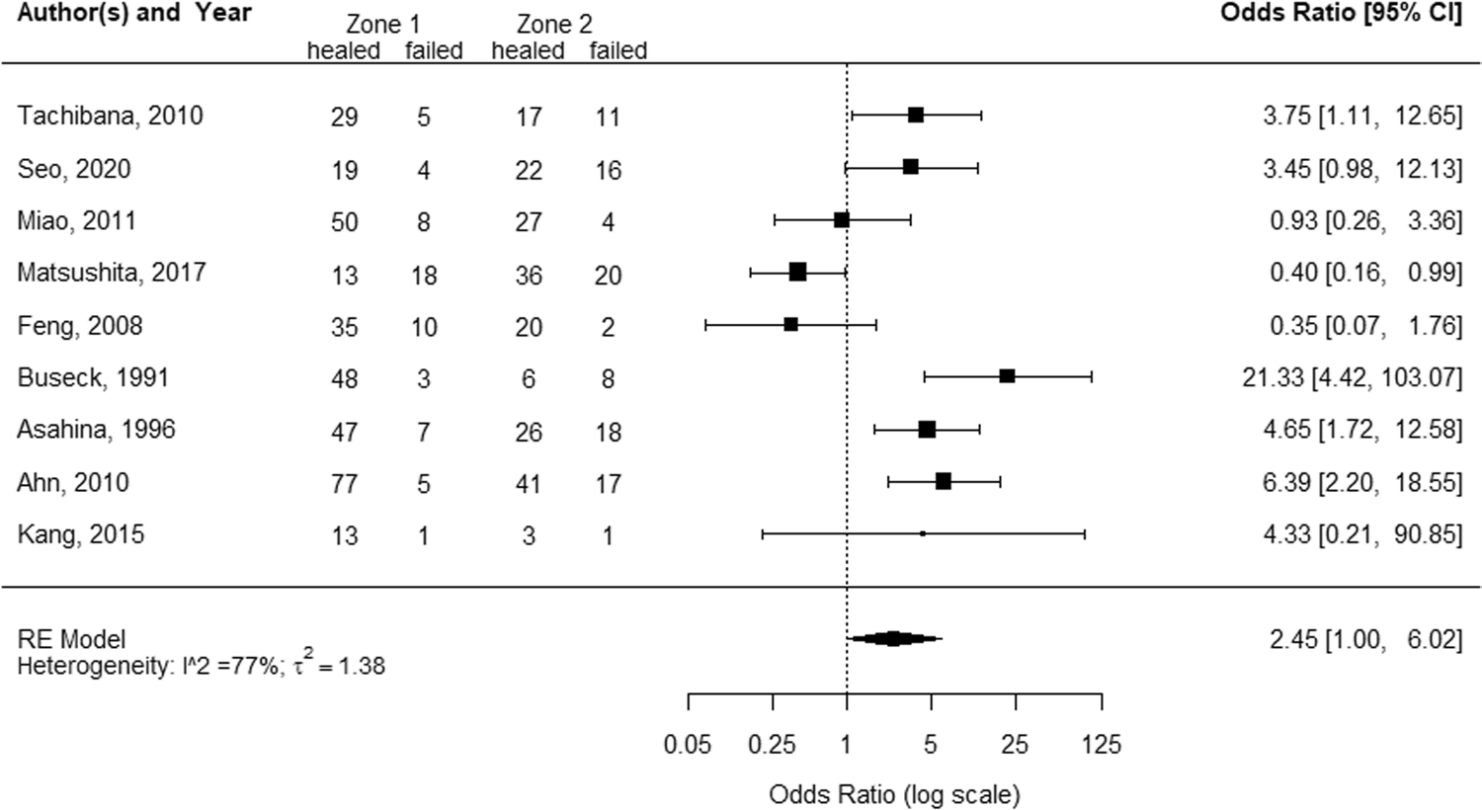
Forest plot for odds ratio of completely healed tears in zone 1 compared with zone 2

The pooled estimated odds ratio of healing in zone 1 compared with zone 2 was 2.5 (95% CI 1.0−6.0), indicating zone 1 tears as 2.5 times more likely to heal than zone 2 tears. Heterogeneity between studies was high (*I*^2^ = 77%) (Fig. 5). The study of Kanto et al. was not included in the meta-analysis for odds ratios since they analyzed healing percentage for “red-white to white-white zones” as one group but did not provide the proportion of healing for the specific “red-white” or “white-white” zone.

## Discussion

The most important finding of the present study was that meniscal tears localized in vascular zone 1 were more likely to heal than tears localized in zone 2. This systematic review and meta-analysis is the first to evaluate the biological healing of meniscal tears using second-look arthroscopy after repair. The pooled healing rate was 83% for tears located in zone 1 and 69% for tears in zone 2. No information on the specific outcome of zone 3 tears was available. The pooled odds ratio for healing was 2.5 when comparing zone 1 and zone 2 tears, suggesting higher healing capacity of zone 1 tears. However, heterogeneity was high and the odds ratio was borderline significant when considering the confidence interval (*I*^2^ = 77%, 95% CI 1.0−6.0). Three studies reported an odds ratio below 1, indicating zone 2 tears as more likely to heal (Fig. 5). Of these studies, only Matsushita et al. show a significant odds ratio (Fig. 5), yet the authors did not provide an explanation for their findings not being in line with the existing literature.

The accuracy of clinical outcomes to assess whether a meniscus is healed is known to be poor [11, 19]. The presence of tibiofemoral joint symptoms does not always distinguish between healed and unhealed meniscal lesions, and is not directly related to biological healing of the meniscus [17]. Biologically healed meniscal tears might result in functionally superior menisci and consequently better long-term outcomes (i.e. compared to incomplete or non-healed tears). Imaging methods are often used to assess whether a meniscal tear has healed. The sensitivity, specificity and accuracy of different imaging modalities such as conventional MRI, (in)direct MR arthrography and CT arthrography to evaluate the healing status of a repaired meniscal tear is higher compared to clinical assessment, but remain limited [16, 19, 26, 27]. Only studies using second-look arthroscopy were included, which is the most reliable technique to assess meniscal healing after repair and remains the “golden” standard [17, 19].

Pre-clinical studies show that peripheral blood supply is needed for meniscal lesions to heal, and tears located in avascular meniscal tissue are unlikely to generate a healing response. It has been discussed that, in the clinical setting, tears located in the outer third of the meniscus are more likely to heal than those in the central thirds due to the avascular nature of the inner meniscus [2, 6, 8, 31, 34, 35]. These findings are comparable to the results of the present study, where tears located in the better vascularized peripheral third are 2.5 times more likely to heal than tears in the middle third. Yeo et al. reviewed the impact of vascularity on meniscal healing after arthroscopic meniscal repair, yet they included studies that used a variety of methods to assess outcome (e.g. reoperation rate, clinical failure, PROMs) and could not make a firm conclusion on vascularity as a predictive factor [36].

Barber-Westin and Noyes focused on tears in the red–white zone and reported a “clinical” healing rate of 83%, where repairs were considered healed when no additional surgery was required and no apparent clinical meniscus symptoms were detected [3]. This is seemingly higher than the healing rate of 69% assessed through second-look arthroscopy. No studies were identified reporting on zone 3 outcomes, which is likely because zone 3 tears are rarely treated with meniscal repair. However, Noyes and Barber-Westin reported that 75% of patients younger than age 20 and 87% of patients older than 40 with tears extending into the avascular zone were asymptomatic for tibiofemoral joint symptoms after meniscal repair at mean follow-up of 51 and 34 months, respectively [22, 23]. It should nonetheless be mentioned that the type of tear might influence the outcome after repair [35]. A radial tear “extending” into the avascular zone might be connected to vascular supply from the peripheral zone, whereas a longitudinal or horizontal tear in zone 3 has no connection with zones 1 or 2.

Vascularization of the meniscus is not the only factor predicting the outcome of meniscal repairs. A wide range of factors have been proposed as predictors of failure rates of meniscal repairs, such as age, tear complexity, sex, body mass index, side of repair, time from injury to surgery, tear length and number of sutures. Of these factors, it has been shown that meniscal repair in combination with ACLR has significantly lower failure rates than meniscal repair alone [28, 30, 31]. To prevent bias and limit clinical heterogeneity, we excluded those studies that did not perform concomitant ACLR. Furthermore, Yeo et al. showed that tear complexity significantly predicted failure rates [36]. There was great cohesion in patient characteristics (i.e. age and gender) among the studies included in this review, increasing comparability between study outcomes. Finally, the pooled outcomes are based on many meniscal repairs, increasing the applicability to the general population of patients with meniscal tears.

The use of the terms “red–red”, “red–white” and “white–white” is discouraged by the International Society of Arthroscopy Knee Surgery and Orthopaedics Sports Medicine (ISAKOS), because the vascular supply of the menisci varies and cannot be precisely determined by rim width alone [1]. However, most of the included studies did use these terms. Vascularity changes throughout life and the degree of vascular penetration in zones 1 and 2 can differ between patients [17]. This meta-analysis determined the effect of meniscal zones on actual healing rate and, indirectly, the effect of vascularity on meniscal healing. Because vascularity is often not directly assessable during surgery, the effect of specific zones on healing rate as described in this review is clinically most useful.

Different surgical techniques have been used (all-inside and inside-out) in the included studies, even within some of the studies themselves. However, the difference in surgical techniques is shown to be associated with minimal change of outcome [10]. Lower limb alignment might be associated with functional outcomes after meniscal repair, but was unavailable in the included studies [36]. Moreover, there was heterogeneity in the patient population, meniscal tear configurations, and postoperative rehabilitation among the included studies. Several comprehensive reviews show minimal difference in healing rate for these various factors [31, 36]. Statistical heterogeneity (*I*^2^) in healing proportion between studies was high (zone 1: 81%; zone 2: 73%; overall: 78%), which should be considered when interpreting the pooled outcomes. Subgroup analyses were performed to explore this heterogeneity, but no direct effect of study-level covariates influencing this heterogeneity could be concluded.

Finally, it was intended only to include studies in which concomitant ACLR was performed. Nevertheless, Feng et al. were also included since they performed concomitant ACLR in all but one single patient. Patients included in this review received different ACL grafts, thereby introducing possible bias. Salem et al. report different failure rates of meniscal repair with concomitant ACLR for different types of ACL grafts [29]. To further identify the influence of the meniscal tear zone on the actual healing of meniscal tears, a large-scale study should be conducted in a more homogenous patient group with a considerable number of tears in all meniscal vascular regions. Knowledge of vascular zone-specific healing rates will guide surgeons in selecting the optimal treatment for individual patients (i.e. surgical repair or partial meniscectomy).

## Conclusion

Healing rates after arthroscopic meniscal repair with concomitant ACLR assessed through second-look arthroscopy were higher for vascular zone 1 than for zone 2. Pooled odds ratio of healing suggested zone 1 tears were more likely to heal than zone 2 tears.

## Appendix I

### Search string PubMed

(“meniscal healing”[tw] OR “meniscus healing”[tw] OR ((“meniscal tears”[tw] OR “meniscal tear”[tw] OR “meniscus tears”[tw] OR “meniscus tear”[tw] OR “meniscal ruptures”[tw] OR “meniscal rupture”[tw] OR “meniscus ruptures”[tw] OR “meniscus rupture”[tw] OR “ruptured menisci”[tw] OR “ruptured meniscus”[tw] OR ((“meniscal”[tw] OR “meniscus”[tw] OR menisc*[tw]) AND (“tears”[tw] OR “tear”[tw] OR tear*[tw] OR ruptur*[tw])) OR “Tibial Meniscus Injuries”[majr] OR “Meniscus/injuries”[majr] OR (“Knee Injuries”[majr] AND “Meniscus”[majr]) OR “meniscal repair”[tw] OR “meniscal repairs”[tw] OR “meniscus repair”[tw] OR “meniscus repairs”[tw]) AND (“Wound Healing”[mesh] OR “healing”[tiab] OR “heal”[tiab] OR “healed”[tiab] OR “failure”[tiab] OR success*[tiab])) NOT (“Animals”[mesh] NOT “Humans”[mesh]) NOT ((“Case Reports”[ptyp] OR “case report”[ti]) NOT (“Review”[ptyp] OR “review”[ti] OR “Clinical Study”[ptyp] OR “trial”[ti] OR “RCT”[ti])) AND english[la]).

### Search string Embase

((“meniscal healing”.ti,ab OR “meniscus healing”.ti,ab OR ((*”knee meniscus rupture”/ OR “meniscal tears”.ti,ab OR “meniscal tear”.ti,ab OR “meniscus tears”.ti,ab OR “meniscus tear”.ti,ab OR “meniscal ruptures”.ti,ab OR “meniscal rupture”.ti,ab OR “meniscus ruptures”.ti,ab OR “meniscus rupture”.ti,ab OR “ruptured menisci”.ti,ab OR “ruptured meniscus”.ti,ab OR ((“meniscal”.ti,ab OR “meniscus”.ti,ab OR menisc*.ti,ab) ADJ5 (“tears”.ti,ab OR “tear”.ti,ab OR tear*.ti,ab)) OR (*”Knee Meniscus”/ AND (“tears”.ti,ab OR “tear”.ti,ab OR “teared”.ti,ab OR ruptur*.ti,ab)) OR “meniscal repair”.ti,ab OR “meniscal repairs”.ti,ab OR “meniscus repair”.ti,ab OR “meniscus repairs”.ti,ab) AND (exp *”Healing”/ OR “healing”.ti,ab OR “heal”.ti,ab OR “healed”.ti,ab OR “failure”.ti,ab OR success*.ti,ab))) NOT (exp “Animals”/ NOT exp “Humans”/) NOT (“Case Report”/ OR “case report”.ti) AND english.la).

### Search string web of science

(TS = (“meniscal healing” OR “meniscus healing”) OR (ts = (“knee meniscus rupture” OR “meniscal tears” OR “meniscal tear” OR “meniscus tears” OR “meniscus tear” OR “meniscal ruptures” OR “meniscal rupture” OR “meniscus ruptures” OR “meniscus rupture” OR “ruptured menisci” OR “ruptured meniscus” OR ((“meniscal” OR “meniscus” OR menisc*) NEAR/5 (“tears” OR “tear” OR tear*)) OR “meniscal repair” OR “meniscal repairs” OR “meniscus repair” OR “meniscus repairs”) AND ti = (“Healing” OR “healing” OR “heal” OR “healed” OR “failure” OR success*)) OR (ti = (“knee meniscus rupture” OR “meniscal tears” OR “meniscal tear” OR “meniscus tears” OR “meniscus tear” OR “meniscal ruptures” OR “meniscal rupture” OR “meniscus ruptures” OR “meniscus rupture” OR “ruptured menisci” OR “ruptured meniscus” OR ((“meniscal” OR “meniscus” OR menisc*) NEAR/5 (“tears” OR “tear” OR tear*)) OR “meniscal repair” OR “meniscal repairs” OR “meniscus repair” OR “meniscus repairs”) AND ts = (“Healing” OR “healing” OR “heal” OR “healed” OR “failure” OR success*))) NOT ti = ”Case Report” AND la = english NOT ti = (“veterinary” OR “rabbit” OR “rabbits” OR “animal” OR “animals” OR “mouse” OR “mice” OR “rodent” OR “rodents” OR “rat” OR “rats” OR “pig” OR “pigs” OR “porcine” OR “horse” OR “horses” OR “equine” OR “cow” OR “cows” OR “bovine” OR “goat” OR “goats” OR “sheep” OR “ovine” OR “canine” OR “dog” OR “dogs” OR “feline” OR “cat” OR “cats”).

### Search string Cochrane

(“meniscal healing” OR “meniscus healing” OR ((“knee meniscus rupture” OR “meniscal tears” OR “meniscal tear” OR “meniscus tears” OR “meniscus tear” OR “meniscal ruptures” OR “meniscal rupture” OR “meniscus ruptures” OR “meniscus rupture” OR “ruptured menisci” OR “ruptured meniscus” OR ((“meniscal” OR “meniscus” OR menisc*) NEAR/5 (“tears” OR “tear” OR tear*)) OR “meniscal repair” OR “meniscal repairs” OR “meniscus repair” OR “meniscus repairs”) AND (“Healing” OR “healing” OR “heal” OR “healed” OR “failure” OR success*))):ti,ab,kw.

## Appendix II

### Modified Newcastle–Ottawa Score

Note: a study can be awarded a maximum of one star for each numbered item within the Selection and Outcome categories. A maximum of two stars can be given for Comparability.

### Selection

#### Representativeness of the exposed cohort

- Truly representative of the average patient with a meniscal tear in the community
- Somewhat representative of the average patient with a meniscal tear in the community
- Selected group of patients, e.g. nurses, volunteers, military personnel
- No description of the derivation of the cohort

#### Selection of the non-exposed (/other vascular zone(s)) cohort

- Drawn from the same community as the exposed cohort
- Drawn from a different source
- No description of the derivation of the non-exposed cohort
- Only one vascular zone described

#### Ascertainment of exposure

- Rim width for classification of vascular region specified (in mm)
- No rim width for classification of vascular region specified (in mm)

#### Demonstration that outcome of interest was not present at start of study

- Yes
- No

### Comparability

#### Comparability of cohorts on the basis of the design or analysis

- Study only includes patients with the same type of tear (longitudinal, bucket-handle, horizontal, complex, etc.)
- Study controls for any other additional factor of possible influence (age, chronicity of tear, tear length, gender, surgical technique, etc.)

### Outcome

#### Assessment of outcome

- Definition of healing based on 2nd-look specified
- Definition of healing based on 2nd-look not specified

#### Was follow-up long enough for outcomes to occur

- Yes, 2nd-look arthroscopy was at ≥ 6 months
- No

#### Adequacy of follow-up of cohorts

- Complete follow-up—all patients received 2nd-look arthroscopy
- Subjects lost to follow-up unlikely to introduce bias, small number lost: > 80% follow-up rate or description provided of those who did not receive arthroscopy
-  < 80% of meniscal repair patients received 2nd-look arthroscopy and no description of those lost
- No statement

## Appendix III

Quality assesment of included studies, using the modified NOS.

**Table:** 

Study	Selection Q1	Selection Q2	Selection Q3	selection Q4	Comparability	Outcome Q1	Outcome Q2	Outcome Q3	Total score
Ahn et al. 2010	B ( +)	A ( +)	A ( +)	A ( +)	A ( +)	A ( +)	A ( +)	C (+ 0)	7
Seo et al. 2020	B ( +)	A ( +)	B (+ 0)	A ( +)	AB ( +)	A ( +)	A ( +)	B ( +)	8
Asahina et al. 1996	B ( +)	A ( +)	B (+ 0)	A ( +)	AB ( +)	A ( +)	A ( +)	B ( +)	8
Buseck et al. 1991	B ( +)	A ( +)	A ( +)	A ( +)	B ( +)	A ( +)	A ( +)	C (+ 0)	7
Feng et al. 2008	B ( +)	A ( +)	B (+ 0)	A ( +)	A ( +)	A ( +)	A ( +)	C (+ 0)	6
Kanto et al. 2019	B ( +)	A ( +)	B (+ 0)	A ( +)	/ (+ 0)	A ( +)	A ( +)	C (+ 0)	5
Matsushita et al. 2017	B ( +)	A ( +)	B (+ 0)	A ( +)	A ( +)	A ( +)	A ( +)	B ( +)	7
Miao et al. 2011	B ( +)	A ( +)	B (+ 0)	A ( +)	A ( +)	A ( +)	A ( +)	B ( +)	7
Tachibana et al. 2010	B ( +)	A ( +)	A ( +)	A ( +)	AB ( +)	A ( +)	A ( +)	B ( +)	9
Kang et al. 2015	B ( +)	A ( +)	B (+ 0)	A ( +)	B ( +)	A ( +)	A ( +)	B ( +)	7

## Appendix IV

1. Ahn JH, Lee YS, Yoo JC, Chang MJ, Koh KH, Kim MH (2010) Clinical and second-look arthroscopic evaluation of repaired medial meniscus in anterior cruciate ligament-reconstructed knees. Am J Sports Med 38(3):472–477
2. Asahina S, Muneta T, Yamamoto H. (1996) Arthroscopic meniscal repair in conjunction with anterior cruciate ligament reconstruction: factors affecting the healing rate. Arthroscopy 12(5):541–545
3. Buseck MS, Noyes FR (1991) Arthroscopic evaluation of meniscal repairs after anterior cruciate ligament reconstruction and immediate motion. Am J Sports Med 19(5):489–494
4. Feng H, Hong L, Geng XS, Zhang H, Wang XS, Jiang XY (2008) Second-look arthroscopic evaluation of bucket-handle meniscus tear repairs with anterior cruciate ligament reconstruction: 67 consecutive cases. Arthroscopy 24(12):1358–1366
5. Kang HJ, Chun CH, Kim KM, Cho HH, Espinosa JC (2015) The Results of All-Inside Meniscus Repair Using the Viper Repair System Simultaneously with Anterior Cruciate Ligament Reconstruction. Clin Orthop Surg 7(2):177–184
6. Kanto R, Yamaguchi M, Sasaki K, Matsumoto A, Nakayama H, Yoshiya S (2019) Second-Look Arthroscopic Evaluations of Meniscal Repairs Associated With Anterior Cruciate Ligament Reconstruction. Arthroscopy 35(10):2868–2877
7. Matsushita T, Nagai K, Araki D, Tanaka T, Matsumoto T, Nishida K, et al. (2017) Factors associated with the status of meniscal tears following meniscal repair concomitant with anterior cruciate ligament reconstruction. Connect Tissue Res 58(3–4):386–392
8. Miao Y, Yu JK, Ao YF, Zheng ZZ, Gong X, Leung KK (2011) Diagnostic values of 3 methods for evaluating meniscal healing status after meniscal repair: comparison among second-look arthroscopy, clinical assessment, and magnetic resonance imaging. Am J Sports Med 39(4):735–742
9. Seo SS, Kim CW, Lee CR, Park DH, Kwon YU, Kim OG, et al. (2020) Second-look arthroscopic findings and clinical outcomes of meniscal repair with concomitant anterior cruciate ligament reconstruction: comparison of suture and meniscus fixation device. Arch Orthop Trauma Surg 140(3):365–372
10. Tachibana Y, Sakaguchi K, Goto T, Oda H, Yamazaki K, Iida S (2010) Repair integrity evaluated by second-look arthroscopy after arthroscopic meniscal repair with the FasT-Fix during anterior cruciate ligament reconstruction. Am J Sports Med 38(5):965–971

## Supplementary Information

Below is the link to the electronic supplementary material.

Supplementary file1 (SAV 10 KB)
